# Biosorption of Cadmium and Lead by Dry Biomass of *Nostoc* sp. MK-11: Kinetic and Isotherm Study

**DOI:** 10.3390/molecules28052292

**Published:** 2023-03-01

**Authors:** Muhammad Kaleem, Lubna Anjum Minhas, Muhammad Zafar Hashmi, Mohammad Ajmal Ali, Rania M. Mahmoud, Saddam Saqib, Moona Nazish, Wajid Zaman, Abdul Samad Mumtaz

**Affiliations:** 1 Department of Plant Sciences, Quaid-i-Azam University, Islamabad 45320, Pakistan; 2 Department of Chemistry, COMSATS University, Islamabad 45550, Pakistan; 3 Department of Botany and Microbiology, College of Science, King Saud University, Riyadh 11451, Saudi Arabia; 4 Department of Botany, Faculty of Science, University of Fayoum, Fayoum 63514, Egypt; 5 State Key Laboratory of Systematic and Evolutionary Botany, Institute of Botany, Chinese Academy of Sciences, Beijing 100093, China; 6 University of Chinese Academy of Sciences, Beijing 100049, China; 7 Department of Botany, Rawalpindi Women University, Rawalpindi 46000, Pakistan; 8 Department of Life Sciences, Yeungnam University, Gyeongsan 38541, Republic of Korea

**Keywords:** biosorption, cadmium, lead, *Nostoc* sp. MK-11, kinetics, isotherms

## Abstract

Cadmium (Cd) and lead (Pb) are global environmental pollutants. In this study, *Nostoc* sp. MK-11 was used as an environmentally safe, economical, and efficient biosorbent for the removal of Cd and Pb ions from synthetic aqueous solutions. *Nostoc* sp. MK-11 was identified on a morphological and molecular basis using light microscopic, 16S rRNA sequences and phylogenetic analysis. Batch experiments were performed to determine the most significant factors for the removal of Cd and Pb ions from the synthetic aqueous solutions using dry *Nostoc* sp. MK1 biomass. The results indicated that the maximum biosorption of Pb and Cd ions was found under the conditions of 1 g of dry *Nostoc* sp. MK-11 biomass, 100 mg/L of initial metal concentrations, and 60 min contact time at pH 4 and 5 for Pb and Cd, respectively. Dry *Nostoc* sp. MK-11 biomass samples before and after biosorption were characterized using FTIR and SEM. A kinetic study showed that a pseudo second order kinetic model was well fitted rather than the pseudo first order. Three isotherm models Freundlich, Langmuir, and Temkin were used to explain the biosorption isotherms of metal ions by *Nostoc* sp. MK-11 dry biomass. Langmuir isotherm, which explains the existence of monolayer adsorption, fitted well to the biosorption process. Considering the Langmuir isotherm model, the maximum biosorption capacity (q_max_) of *Nostoc* sp. MK-11 dry biomass was calculated as 75.757 and 83.963 mg g^−1^ for Cd and Pb, respectively, which showed agreement with the obtained experimental values. Desorption investigations were carried out to evaluate the reusability of the biomass and the recovery of the metal ions. It was found that the desorption of Cd and Pb was above 90%. The dry biomass of *Nostoc* sp. MK-11 was proven to be efficient and cost-effective for removing Cd and especially Pb metal ions from the aqueous solutions, and the process is eco-friendly, feasible, and reliable.

## 1. Introduction

Heavy metal pollution is a severe global issue because of its non-biocompatibility, concealment, and high toxicity [1]. In general, mining, metal manufacturing, electronic electroplating, agricultural activities, and wastewater discharge from metallurgy are the major anthropogenic factors that contribute to the rising level of harmful metals in the environment [2,3,4]. A long-term exposure to heavy metals can cause irreversible damage to many biological systems [5].

Cd and Pb are considered the most lethal environmental pollutants because they are not only dangerous to plants but also to humans [6]. Their pollution is a serious environmental issue that harms aquatic ecosystems and human health [7]. The battery, paint, pigment, fertilizer, and refinery industries account for most of the Cd pollution, and exposure to this hazardous metal can cause cancer and kidney issues in humans [8]. Furthermore, exposure to Cd may also have hypertension, renal failure, teratogenic, lung damage, and hepatic injury consequences [9]. On the other hand, steel and alloys, the plastics industry, paints, pigments, cables, batteries, and glass are the main sources of Pb pollution [10]. Pb is considered a cumulative toxin and is deemed hazardous in high quantities. Its toxicity can damage the gastrointestinal tract, immune system, kidneys, liver, and circulatory system [11,12].

There have been numerous attempts to investigate the methods for cleaning up heavy metal-polluted water, including conventional chemical and physical techniques such as membrane filtration, reverse osmosis, electrolysis, chemical precipitation, coagulation, and ion exchange [13]. However, occasionally, the viability of such methods is constrained by technological or economic constraints. Biosorption has been recognized as one of the most attractive and successful methods for heavy metal removal; it has several advantages, such as its versatility, high efficiency, simple operation, and low production of harmful by-products and intermediates [14]. Biomasses have long been regarded as spectacular adsorbents due to their strong metal chelating capability and abundance as natural renewable resources [15]. Many biological substances, including bacteria, fungi, and algae, have demonstrated excellent potential for the remediation of toxic heavy metals [16,17]. Among the biomaterials, cyanobacteria are one of the biological materials that have the capability of removing heavy metals from wastewater [18,19,20,21]. Cyanobacteria have special advantages over other microorganisms, which are associated with their increased volume of mucilage with strong binding affinities, increased surface area, and simple nutritional requirements [22]. Notably, cyanobacteria are expected to be productive in heavy metal removal due to their rich binding sites and high binding affinity to heavy metals. For instance, cyanobacteria that produce exopolysaccharides (EPS) have been studied as chelating agents for the removal of Pb, Cr, and Cd ions from aqueous solutions [23,24]. According to the literature, there has been no investigation into the cadmium and lead biosorption using this cyanobacterium (*Nostoc* sp. MK-11) biomass. Moreover, selected biomass was used as a biosorbent in this investigation due to being renewable, low cost, and natural.

The current research looks into the potential use of *Nostoc* sp. MK-11 dry biomass for the removal of Pb and Cd ions from synthetic aqueous solutions. Experimental conditions such as the contact time, pH, initial metal concentrations, and biomass dosage were studied as biosorption-affecting parameters. Mechanisms of biosorption were investigated in terms of kinetics. Langmuir, Freundlich, and Temkin isotherms were applied to evaluate the biosorption equilibrium data. Moreover, a desorption study was carried out for the recovery of Pb and Cd and the reusability of biomass.

## 2. Results and Discussion

### 2.1. Identification of the Nostoc sp. MK-11

**Figure 1:**
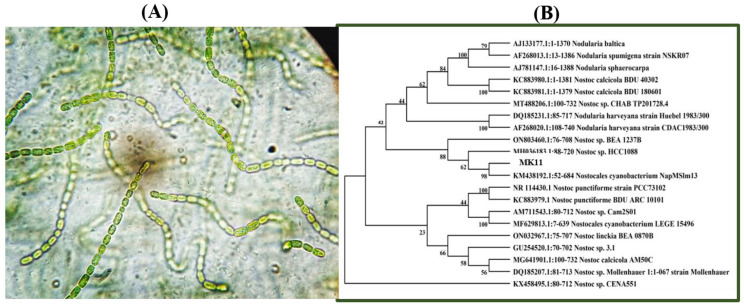
(**A**) Micrograph of cyanobacterium *Nostoc* sp. MK-11, (**B**) phylogenetic tree based on the 16s rRNA sequences of *Nostoc* sp. MK-11.

The *Nostoc* sp. MK-11 was originally isolated from the polluted aqueous environment of Islamabad, the capital of the Islamic Republic of Pakistan. The observed morphology (Figure 1A) and BLAST-based NCBI search with 100 percent similarity and phylogenetic analysis (Figure 1B) showed that the species MK-11 belongs to the genus *Nostoc*. All these observations and analyses confirmed the identification of the studied cyanobacterium as *Nostoc* sp. MK-11. It has been investigated in terms of its Cd and Pb ions biosorption and desorption potential, in addition to its kinetic and isothermal behavior during the biosorption processes.

### 2.2. Characterization

#### 2.2.1. FTIR

**Figure 2:**
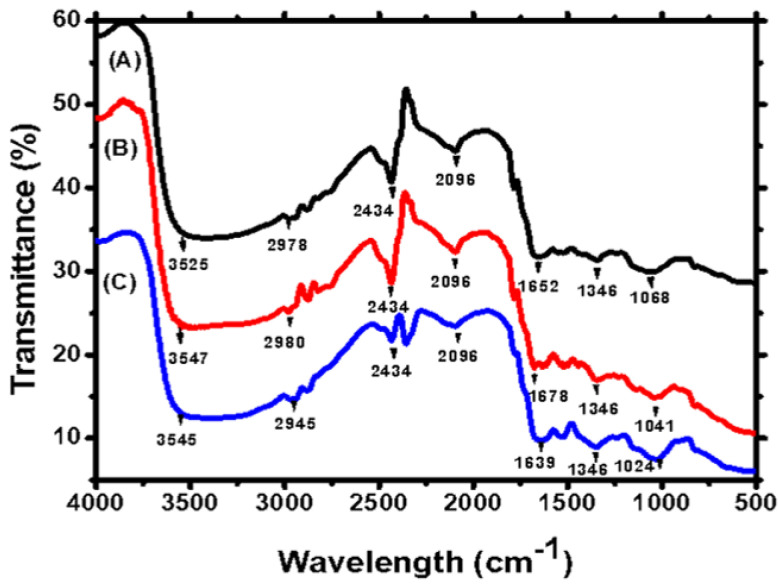
FTIR spectra of (A) non-treated and (B) Cd—and (C) Pb—treated dry biomass.

Figure 2 depicts the FTIR spectra of *Nostoc* sp. MK-11 biomass before and after the biosorption of the Pb and Cd ions. The surface of the biosorbent consisted of numerous functional groups. The core absorption bands in the cyanobacterial biomass at 3525 cm^−1^ are related O-H and N-H stretching in the hydroxyl and amine functional groups, respectively. The stretching distribution of CH_2_ and CH_3_ is displayed by the bands between 2800 and 3000 cm^−1^ of the symmetric and asymmetric aliphatic chain (C-H) stretching vibrations. The band at 1652 cm^−1^ showed the C=O functional group’s asymmetric vibration and the presence of the SO (sulfoxide group). In general, biomass characterization is helpful for deciphering the process of metal binding on the surface of biomass and assessing the efficacy of biosorbents for use in the biosorption process [25]. The FTIR results suggest that the dry biomass of *Nostoc* sp. MK-11 is useful for the biosorption of Cd and Pb ions. Figure 2 depicts peak shifting after Cd and Pb ions biosorption. The shifting of the peaks related to functional groups such as hydroxyl, amines, sulfoxide, and carbonyl is an indication of an interaction between metals and the biomass functional groups, which confirms the involvement of these functional groups in the removal of Pb and Cd ions from the aqueous solution. Similar FTIR results were described in the previous studies, which presented the involvement of functional groups in the removal of metals by the dry biomass of algae [26,27,28,29].

#### 2.2.2. SEM

**Figure 3:**
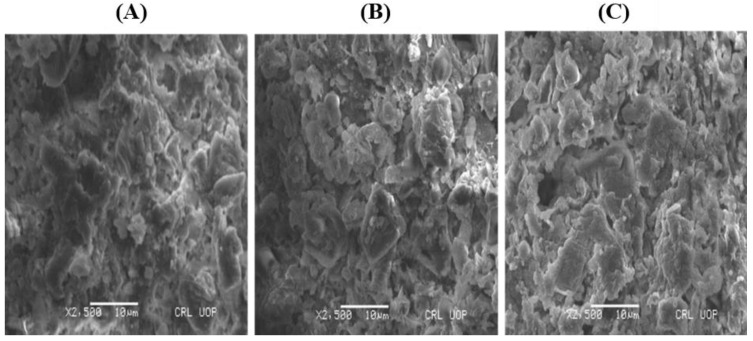
SEM micrographs of *Nostoc* sp. MK-11 (**A**) raw biomass, (**B**) Cd-loaded biomass, (**C**) Pb-loaded biomass.

The morphology of non-treated and Cd and Pb treated biomass was investigated using SEM. As shown in Figure 3A, the biomass surface appeared dull and smooth before the biosorption of Cd and Pb ions. However, the biosorbent surface changed after the biosorption process (Figure 3B,C); it became brilliant, rough, and sharp ended. These observed changes in the biomass morphology were caused by the precipitation of Cd and Pb ions onto the surface of the dried biomass. Similar results were described for *Parachlorella* sp. when Cd was removed from the aqueous solution [30]. The changes in the morphology of the biomass may be credited to the cross-linking of functional groups and metal ions [25].

### 2.3. Effect of pH

**Figure 4:**
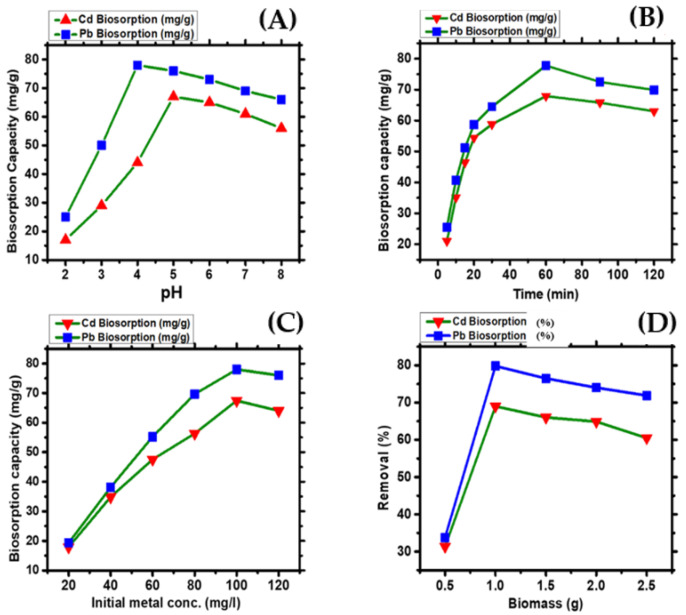
(**A**) Effect of pH, (**B**) contact time, (**C**) initial metal concentrations, (**D**) biomass dosage on the biosorption of Cd and Pb ions.

The biomass of algae possesses a rich carboxyl group content from guluronic and mannuronic acids on the polysaccharide cell wall, which suggests that variation in the pH of the solution could affect the biosorption [31]. Figure 4A depicts the pH effect on the biosorption of Pb and Cd ions onto *Nostoc* sp. MK-11 dry biomass. The highest biosorption was found at pH 5 for Cd and at pH 4 for Pb. Therefore, all further experiments were conducted at pH 4 and 5. Metal biosorption began to decline when the pH level crossed from 5 for Cd and 4 for Pb, as shown in Figure 4A. At a low pH (2–3), a rise in the positive charge on the biomass surface restricted the metal cations approach because of the repulsion force. A decrease in the biosorption at a low pH was due to a higher number of hydrogen ions, which form ligands with metal ions at the biosorbent surface [32]. According to the findings of this study, the sorption of Cd and Pb ions occurred at two different pH values, which is due to differences in the metal properties such as the electronegativity, size, or the availability of extra metal ions.

### 2.4. Effect of Contact Time

One of the most essential components in the biosorption process is contact time. Figure 4B represents the biosorption of Cd and Pb ions as a function of the contact time and describes how biosorption increases up to 60 min contact time. After that, a gradual decrease in metal ion biosorption occurred. The decrease in the biosorption after the 60 min contact time may be due to the discharge of metal ions from the surface of the biomass into the solution [33]. Consequently, 60 min was chosen as the optimum contact time for further investigations. The findings of our study are consistent with those of an earlier study in which the maximum biosorption of lead and cadmium occurred at 60 min of contact time [34,35]. 

### 2.5. Effect of Various Initial Metal Concentrations

Figure 4C represents the effects of initial metal concentrations (ranging from 20 mgL^−1^ to 120 mgL^−1^) on the biosorption of Pb and Cd ions by dry *Nostoc* sp. MK-11 biomass. The biomass showed an improved ability to absorb Cd and Pb ions up to 100 mgL^−1^. The rise in the metal concentrations enhances the metal ions driving force from the bulk solution to the biomass and improves the biosorption capacity [29]. However, at 120 mgL^−1^ of metal ion concentrations, a slight decrease in the biosorption capacity of the biomass occurred. It might be brought on by more metal ions remaining unabsorbed in the solution as the biomass binding sites become saturated at higher ion concentrations. This agrees with the results of previously described studies [36,37]. 

### 2.6. Effect of Biomass Dosage

The biomass dosage effect was investigated using biomass in the range of 0.5 to 2.5 g L^−1^ (Figure 4D). The results show that metal ion biosorption hinges on the dosage of biomass in the solution. Maximum biosorption of the Cd and Pb ions were obtained at 1 g/L of biomass in solution, and the biosorption efficiency decreased when the concentration of the biomass was more than 1 g L^−1^. This might be because of aggregate formation at an increased biomass dosage, which ultimately decreases the surface area and metal binding sites. This finding is similar to the results of a previously described study in which the biosorption of metals enhanced as the dosage of the biomass increased [38,39].

### 2.7. Kinetic Study

**Figure 5:**
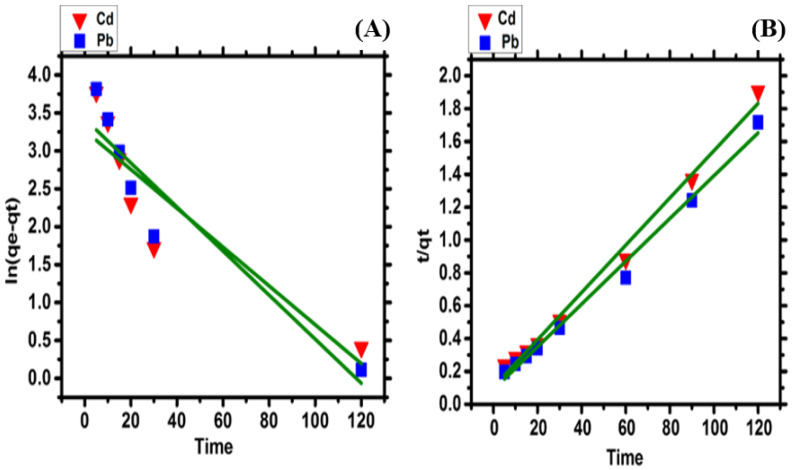
Kinetic study (**A**) pseudo-first order, (**B**) pseudo-second order, models for biosorption of Pb and Cd ions using dry *Nostoc* sp. MK-11 biomass.

It is quite interesting to use experimental data in kinetic studies to figure out mechanisms, such as chemical reactions and mass transfer, that influence the rate of biosorption [40,41]. To study the kinetics of biosorption, pseudo-first, and pseudo-second order models, (Figure 5A,B) were applied.

Based on the calculated qe, experimental qe, and correlation coefficients (R^2^), the rate of the Pb and Cd ions’ biosorption was found to be well suited to pseudo-second order kinetics. The qe obtained from the pseudo-first order does not fit well to the qe experimental and the R^2^ values are also not up to the mark (Table 1), which made it clear that the pseudo-first order does not fit to the biosorption data. Previous studies also showed similar results [42]. In pseudo-second order kinetic modelling, the experimental qe agree with the qe calculated, and R^2^ is quite close to unity, as described in Table 1. It indicates that pseudo-second order is well suitable to the biosorption of Pb and Cd ions. The well-fitting of pseudo-second order shows that the rate of biosorption is limited by chemical sorption [41,43]. Previous studies used the dry biomass of *Chlamydomonas reinhardtii* and *Oedogonium* sp. to characterize the Cd and Pb biosorption kinetics using pseudo-second order [35,44].

### 2.8. Isotherm Study

**Figure 6:**
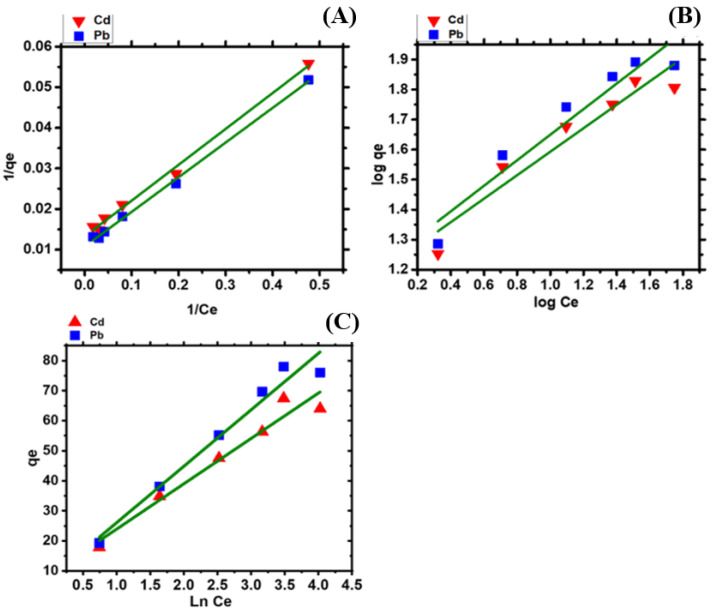
(**A**) The Langmuir, (**B**) Freundlich isotherm, (**C**) Temkin isotherm for biosorption of Cd and Pb ions from aqueous solutions using *Nostoc* sp. MK-11 biomass dry biomass.

The isotherm represents the correlation between the metal ion concentrations in the solution at equilibrium and the concentrations of metal ions adsorbed on the surface of the biomass. In the current study, three equilibrium isotherm studies, Langmuir, Freundlich, and Temkin, were performed (Figure 6A–C). 

Table 2 shows the computed parameters of isotherm models as well as their R^2^ coefficient of correlation. Consequently, the R^2^ values confirmed that the biosorption data followed Langmuir’s isotherms. Langmuir’s isotherm model calculated the q_maxas_ as 75.757 and 83.963 mg/g for Cd and Pb ions, respectively, which corresponded to the experimental qe 67.45 for Cd and 78 mg/g for Pb. The biosorption of Pb and Cd ions by *Nostoc* sp. MK-11 dry biomass took place as a homogeneous monolayer on the dry biomass surface [45,46]. Filamentous algae from the Chlorophyta (*Cladophora calliceima*, *Pithophora oedogonium*, *Hydrodictyon reticulatum*, and *Spirogyra neglecta*) and cyanobacteria (*Aulosira fertilissima*) non-living biomass have shown Langmuir isotherm fitting for Cd and Pb biosorption [32].

### 2.9. Desorption

It is important to assess the reusability of dried biomass to ensure that the biosorption process is economical. To determine the ratio of desorption to biosorption (D/B), three cycles of sorption and desorption tests were carried out. Changes in the desorption to biosorption ratio were used to evaluate the likelihood of reusing the dry biomass [47,48]. The metal biosorption results indicated that the *Nostoc* sp. MK-11 biomass was efficient for the biosorption of Pb and Cd ions. Using 0.1 M HCL, over 90% of both metals underwent desorption even though the sorption capability of the cyanobacterial biomass decreased after the first cycle (Table 3).

The metal binding regions in the cyanobacterial cells may have been destroyed by the contaminated solution, which decreased the biosorption effectiveness during sorption and desorption. However, by using a suitable chemical agent, the process of regeneration might be enhanced without reducing the potential of the biosorbent [49]. Both the loss of functional groups and the mass of the biosorbent were most likely responsible for the abrupt drop. The surface functional groups of the biomass have been found to be destroyed by high acid solutions, which disrupted the process of desorption. However, it is important to carry out the desorption because it enables the recovery of absorbed metals using an easy approach (such as electrolysis) for other applications [50,51]. This demonstrated that *Nostoc* sp. MK-11 have the potential to be used as a dual agent for heavy metal removal from the environment and heavy metal recovery from biosorbent for useful industrial applications.

## 3. Materials and Methods

### 3.1. Cyanobacterial Strain and Biomass 

The cyanobacterium, *Nostoc* sp. MK-11, was isolated from the polluted aqueous environment of Islamabad, Pakistan. The BG11 solid media [52] was prepared with 1.5% agar. In total, 20 mL of this media was poured in each Petri plate and allowed to solidify under UV illumination in the Laminar flow hood. A homogenized sample of approximately 500 μL was poured in each Petri plate and gently spread with a glass rod. The samples of inoculated Petri plates were stored at 25 ± 2 °C under continuous LED white light illumination with a 2000 lux intensity. After 10 days, the agar plates were observed under a light microscope and colonies were picked and reinoculated onto new agar Petri plates. After carefully picking, the axenic culture was cultivated in test tubes and conical flasks containing BG11 liquid media.

Morphological and molecular analysis were used for the identification of the isolated cyanobacterium. Morphological characters were observed using light microscopy. For a molecular identification, the genomic DNA was extracted using the CTAB method and a mortar and pestle. The sequences of the PCR-amplified DNA fragments encoding 16S ribosomal RNA region were achieved using cyanobacteria specific primers CYA 106 F as a forward primer and CYA 781 R (a) as a reverse primer [53]. NCBI BLAST-based similarity search and phylogenetic analysis were carried out during the molecular identification. Phylogenetic analyses were performed through the neighbor-joining method using MEGA-X software [54,55].

For producing biomass, the axenic cultures of the cyanobacterium were cultivated in multiple 100, 250, and 500 mL Erlenmeyer flasks containing 70, 200, and 350 mL of BG-11 media, respectively [52]. The cultures were kept at 25 ± 2 °C under 2000 lux white LED light. The cultures were harvested at the maximum growth by centrifugation at 4000 rpm for 15 min. After washing with double distilled water, the pellet was left to air dry. The dry biomass was crushed finely and sieved through a 100 μm mesh to achieve the amorphous state. The powdered form was then stored in a sealed bottle to avert rehydration.

### 3.2. Batch Experiments

In batch experiments, the effects of initial metal concentrations, solution pH, contact time, and biomass dose on the biosorption of the Pb and Cd ions from the synthetic aqueous solutions were studied. The effect of pH was studied with a contact time of 60 min, 1 g L^−1^ of biomass, and concentrations of metals 100 mg L^−1^. The optimal contact time was found at 100 mg L^−1^ of metal concentrations, pH 4 and 5 for Pb and Cd, respectively, and 1 g L^−1^ of biomass. By adding NaOH or HCL, the liquids’ initial pH was adjusted, and during the experiments, the pH of the solutions was not controlled. After optimizing the pH and contact time, the effect of the initial metal concentrations with a range of 20–120 mg L^−1^ was studied at 60 min contact time, 1g L^−1^ biomass at pH 4 and 5 for Pb and Cd ions, respectively. In the last study, the biomass impact on the biosorption of Pb and Cd ions was investigated at metal concentrations of 100 mgL^−1^, pH 4 and 5, and contact time of 60 min.

Batch studies were conducted with 100 mL of 100 mg L^−1^ Pb and/or Cd ion concentrations in 250 mL Erlenmeyer flasks at pH 4 and 5 and mixed with a specific quantity of cyanobacterial biomass. Metals and biomass mixtures were stirred at 150 rpm using an orbital shaker for a predetermined duration of the contact time at 25 °C with three replications. The final and initial metal concentrations of the solutions were determined using a flame atomic absorption spectrometer (FAAS) (Agilent, Santa Clara, CA, USA) after the suspension’s filtration with Whatman 40 ash-free paper. Equations (1) and (2) were used to calculate the biosorption capacity (mg/g) and efficiency (%) of biomass, respectively.

(1) $$q_{e}=\frac{\left(C_{i}-C_{e}\right)}{W}\times V$$

(2) $$P=\frac{\left(C_{i}-C_{e}\right)}{C_{i}}\times 100\;$$

*C_i_* represents the initial concentration of the metals, *C_e_* is the metal concentrations at equilibrium (mg L^−1^), *V* represents the volume of the solution (L), and *W* shows the weight of the biomass (g).

### 3.3. Biosorbent Characterization

Dry cyanobacterial biomass (before and after biosorption) was characterized using Fourier transform infrared spectroscopy (FTIR) and scanning electron microscope (SEM). The surface functional groups of the biomass were identified through FTIR [56], and the morphology of the raw and metal-treated biomass was investigated using SEM (JSM5910 JEOL, Tokyo, Japan).

### 3.4. Kinetics Study

The biosorption rate of the Pb and Cd ions onto the *Nostoc* sp. MK-11 dry biomass was studied using pseudo-first and pseudo-second order kinetic models. Equation (3) represents the pseudo-first order as follows:

*ln*(*qe* − *qt*) = *lnqe* − *K*_1_*t* (3)

*K*_1_ is a pseudo-first order equilibrium rate constant, and *qt* is the biosorption capacity at time *t*.

The pseudo-second order is described in Equation (4) as follows:

(4) $$\frac{t}{q_{t}}=\frac{1}{K_{2q_{e}^{2}}}+\frac{1}{q_{e}}t$$

*K*_2_ is the pseudo-second order rate constant determined by plotting *t*/*q_t_* vs. *t*.

### 3.5. Isotherm Study

The processes of the adsorption isotherm’s features were assessed using the Langmuir, Freundlich, and Temkin isotherm models. The parameters of the isotherms were determined using the following equations:

Langmuir isotherm equation:

(5) $$\frac{1}{q_{e}}=\frac{1}{K_{L}^{\;\;\;\;\;\;\;\;}q_{max}}\cdot \;\frac{1}{C_{e}}+\frac{1}{q_{max}}$$

(6) $$R_{L}=\frac{1}{1+C_{i}\times K_{L}}$$

*K_L_* is Langmuir’s isotherm constant, *q_max_* is the maximum adsorption capacity, and *R_L_* is the separation factor.

Freundlich isotherm equation:

(7) $$Logq_{e}=LogK_{f}+\frac{1}{n}logC_{e}$$

*K_f_* shows the Freundlich’s constant, and *1*/*n* is the adsorption intensity.

Temkin isotherm equation:

(8) $$qe=\frac{RT\;\;}{b_{T}}\ln\left(A_{T}\;C_{e}\right)$$

(9) $$qe=B_{T}\;ln\;\left(AT\right)+B_{T}\;ln\;(C_{e})$$

(10) $$B_{T}=\frac{RT}{b_{T}}$$

*A_T_* is the Temkin equilibrium binding constant, *b_T_* shows the Temkin isotherm constant, *R*^2^ is the coefficient of the correlation constant, *B* is a constant related to the heat of sorption, and *T* is the temperature.

### 3.6. Desorption Study

To discover the reusability potential of the biomass, biosorption–desorption experiments were carried out three times using the same preparations of dried biomass. In total, 0.1 M of hydrochloric acid (HCL) was used for the desorption of the metals. The desorption potential was investigated by shifting metal-loaded biomass to the desorption medium, and this was shaken at 100 rpm for 1 h at 25 °C. After each cycle of sorption and desorption, saline solution was used to wash and recondition the biomass [35]. FAAS was used to calculate the final concentration of metals in the aqueous phase, as explained above. The quantity of adsorbed metal ions onto the biomass surface and the final metals concentration in the sorption medium were used to compute the desorption ratio. Equation (7) was used to evaluate the efficiency of desorption (ED).

(11) $$\mathrm{ED}=\frac{q_{D}}{q_{B}}\;\times \;100$$

*q_D_* is the quantity of desorbed metal ions and *q_B_* is the amount of adsorbed metal ions.

### 3.7. Data Analysis

**Figure 7:**
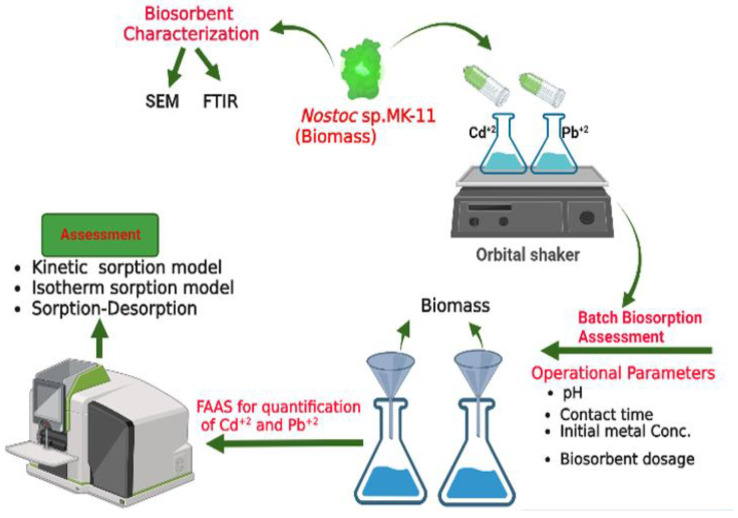
Pictorial representation of batch manner experiments to evaluate the biosorption of Pb and Cd ions using *Nostoc* sp. MK-11 as a biosorbent.

OriginPro 8.5 and Microsoft Office Excel (2010) software were used for the data analysis. The results were described as the mean of 3 (*n* = 3) replicates. The biosorption of the Pb and Cd ions employing *Nostoc* sp. MK-11 as a biosorbent is illustrated in a series of batch studies (Figure 7).

## 4. Conclusions

Cadmium and lead are environmental and industrial heavy metal pollutants classified as human carcinogens. Using *Nostoc* sp. MK-11 dry biomass as a biosorbent, batch biosorption experiments were conducted to optimize the conditions for the removal of Pb and Cd ions from synthetic aqueous solutions. The optimum conditions obtained for the maximum removal of Cd and Pb ions were initial metal concentrations of 100 mg L^−1^, *Nostoc* sp. MK-11 dry biomass of 1 g L^−1^, a contact time of 60 min, and pH 4 and 5 for Pb and Cd, respectively. The primary groups involved in the biosorption of Cd and Pb ions have been identified as hydroxyl, amines, sulfoxide, and carbonyl. After the biosorption process, SEM micrographs of the *Nostoc* sp. MK-11 biomass show that it can adsorb and remove Pb and Cd ions from an aqueous environment. The biosorption of Cd and Pb ions onto *Nostoc* sp. MK-11 dry biomass followed the pseudo-second order kinetic model. The Langmuir isotherm mode was well fitted to biosorption equilibrium, which showed the homogenous monolayer biosorption of both metals onto the dry biomass of *Nostoc* sp. MK-11. The biomass maximum biosorption capacities were calculated as 75.757 and 83.963 mg^−1^ for Cd and Pb ions, respectively, by the Langmuir isotherm model. The desorption of Pb and Cd from the *Nostoc* sp. MK-11 dry biomass was greater than 90% using 0.1 M of HCL. The approach adopted for the biosorption of Cd and Pb is affordable, eco-friendly, practical, and safe. The dry biomass of *Nostoc* sp. MK-11 might be used as a cheap and efficient biosorbent for the removal of Cd and Pb ions from contaminated water.

## Figures and Tables

**Table 1:** Pseudo-first and pseudo-second order kinetics.

	qe (Experiment)	Pseudo-First Order			Pseudo-Second Order		
Metals	(mg/g)	qe _(cal)_ (mg/g)	K_1_ (min^−1^)	R^2^	qe_(cal)_ (mg/g)	K_2_ (gmg^−1^ min^−1^)	R^2^
Cd	67.9	26.206	−0.0004	0.770	69.492	0.00196	0.991
Pb	77.75	30.569	−0.0003	0.863	76.923	0.00192	0.990

**Table 2:** Langmuir isotherm Freundlich isotherm and Temkin parameters MK11.

	Langmuir				Freundlich			Temkin		
Metals	q_max_ (mg/g)	K_L_ (L/mg)	R_L_	R^2^	K_F_ (mg/g)	1/n	R^2^	K_T_	B_T_	R^2^
Cd	75.757	0.149	0.0627	0.993	15.915	0.391	0.897	1.752	15.796	0.947
Pb	83.963	0.428	0.0098	0.998	27.664	0.328	0.844	7.228	14.68	0.928

**Table 3:** Sorption–desorption of metals.

Metals	Cycles	Biosorption (S) (mg/g)	Desorption (D) (mg/g)	Ratio of D/B (mg/g)	% Desorption
Cd	1	65.65	61.22	0.932	93.252
	2	62	57.5	0.927	92.741
	3	57.02	52.11	0.913	91.388
Pb	1	74.49	71.4	0.958	95.851
	2	69.22	64.45	0.931	93.108
	3	60	56	0.933	93.333

## Data Availability

The data that support the findings of this study are available from the corresponding author (A.S.M) upon request.

## Sample Availability

Samples of the compounds are not available from the authors.
